# Differences between diaphragmatic compound muscle action potentials recorded from over the sternum and lateral chest wall in healthy subjects

**DOI:** 10.1038/s41598-022-11930-1

**Published:** 2022-05-27

**Authors:** Gihan Younis, Noha El Sawy, Rehab Elnemr, Doaa Madkour

**Affiliations:** Physical Medicine, Rheumatology and Rehabilitation Department, Faculty of Medicine, Alexandria University, El-Khartoom Square, Alexandria, 21526 Egypt

**Keywords:** Neuroscience, Neurology

## Abstract

To report normative data for diaphragmatic compound muscle action potentials (DCMAPs) recorded from over the sternum and lateral chest wall (LCW) and highlight factors that may contribute to variations in DCMAP parameters at the two sites. The phrenic nerve of seventy-three healthy subjects was bilaterally stimulated at the posterior border of the sternocleidomastoid muscle. DCMAPs from over the sternum and LCW were recorded (inspiration/expiration). Normative values of sternal and LCW DCMAPs were presented. The mean values of latency of LCW DCMAPs, duration of sternal DCMAPs and area from both recording sites are close to values reported by other studies. The mean values of latency of sternal DCMAPs are higher than that reported by other studies. Significant differences were found between sternal and LCW potentials in the mean latency, amplitude, and area (*p* < 0.001). The duration did not differ between the two sites. Differences were found between inspiration and expiration, right and left sides, and men and women. Regression analysis showed a relation between latency of sternal and LCW potentials and age. Latency (LCW potentials) and amplitude and area (sternal/LCW potentials) were related to gender. Amplitude (LCW potentials/inspiration) and area (sternal potentials/inspiration) were related to chest circumference (*p* = 0.023 and 0.013 respectively). Area (sternal potentials/expiration) was related to the BMI (*p* = 0.019). Our normative values for sternal and LCW DCMAPs are provided. Notable differences in the DCMAPs parameters were detected between the two recording sites, inspiration and expiration, right and left, and men and women. The technique of phrenic nerve should be standardized.

## Introduction

Phrenic nerve conduction study has found increasing application in the diagnosis of phrenic neuropathy associated with different medical and surgical conditions^1–12^. Phrenic nerve conduction study provides a sensitive indicator of phrenic nerve function when the nerves are affected by either local lesions or by generalized neuropathies. Assessment of phrenic nerve function is required in the study of patients with diaphragmatic weakness and candidates for permanent diaphragm pacing^13^. Many studies found abnormalities in phrenic nerve conduction in patients with chronic obstructive pulmonary diseases^14^.

Percutaneous electric stimulation of the phrenic nerve at the neck with recording of the diaphragmatic compound muscle action potential (DCMAP) by surface electrodes is easy, quick, and non-invasive technique^2^. Amplitude, latency, area, and duration are measures used to evaluate phrenic nerve integrity^15–18^.

Several studies have been conducted in healthy individuals to describe diaphragmatic potentials and normative values have been provided. The influence of age, gender, height, body mass index (BMI), chest circumference, and other subjects’ characteristics were shown^15–18^. Values during inspiration and expiration were also provided^15–17^.

The phrenic nerve can be stimulated at the supraclavicular fossa^15–17,19,20^ or at the posterior border of the sternocleidomastoid muscle at the level of the cricoid or thyroid cartilage^21–23^. DCMAPs are commonly recorded from over the sternum with the active electrode (G1) fixed 5 cm above the xiphoid process and the reference electrode (G2) placed 16 cm from G1 on the chest margin ipsilateral to the stimulated phrenic nerve^15–18^ or from over the lateral chest wall (LCW) with recording electrodes placed in the 6th, 7th, or 8th intercostal space along the anterior axillary line^11,21,23–26^. Diaphragmatic potentials are less commonly recorded from the 7th, 8th, or 9th intercostal space on the anterior or anterolateral chest^27,28^.

A number of studies demonstrated that different positions of the recording electrodes contribute to variability of phrenic nerve conduction study parameters. Swenson and Rubenstein^19^ found significant differences in the amplitude of potentials recorded from over the xiphoid and lateral chest wall. Dionne et al.^20^ recorded diaphragmatic potentials from over six different recording sites and reported similar finding. McKenzie and Gandevia^22^ found variation in the latency of DCMAP recorded at different sites. They concluded that recording electrodes at each site are relatively selective for the adjacent portion of the diaphragm^22^ that is innervated by separate nerve branches^29^.

The results from these studies are significant when evaluating segmental lesions of diaphragmatic innervation resulting from different medical or surgical conditions^22^. These studies were however conducted on a limited number of healthy individuals (20, 11, and 3 subjects) and did not define reference values for DCMAP parameters at different sites^19,20,22^.

## Aim of the work

The aim of this study was to report normative data for DCMAP parameters recorded from over the sternum (sternal DCMAP) and lateral chest wall (LCW DCMAP) in healthy individuals and highlight factors that may contribute to variations in the measurements at the two sites.

## Subjects and methods

Seventy-three healthy individuals (48 men and 25 women) participated in the study. Their age ranged from 18 to 61 years (mean: 37.66 ± 11.51) with almost equal number of subjects in each decade. Subjects were recruited from hospital personnel (students, employees, and relatives of patients). None of the subjects was current or ex-smoker (to avoid the impact of smoking on the lungs i.e. chronic obstructive pulmonary disease or emphysema) and none was involved in any sport activity. Exclusion criteria included pregnancy, chest wall abnormalities, and the presence of any medical condition.

Their mean chest circumference (measured at the fourth intercostal space/auxiliary level)^30^ was 93.96 ± 7.77 cm, height was 1.69 ± 0.08 m, and weight was 72.84 ± 12.95 kg. The mean BMI was 25.73 ± 5.08 kg/m^2^. Two subjects (2.9%) were underweight, thirty-two (45.7%) had normal weight, twenty-four (34.3%) were overweight, and twelve (17.1%) were obese.

The study was approved by the local ethics committee of the Faculty of Medicine, Alexandria University and an informed written consent was obtained from all participants.

### Phrenic nerve conduction study

Electrophysiological studies were conducted on Nicolet VikingQuest version 11 USA electromyography machine. During electrophysiological examination, the skin was kept warm around 32-34 °C. A bipolar surface stimulator was used to provide stimulation. Self-adhesive surface electrodes were used for recording. The ground electrode was a disc electrode placed between the stimulating and the recording electrodes.

**Figure 1:**
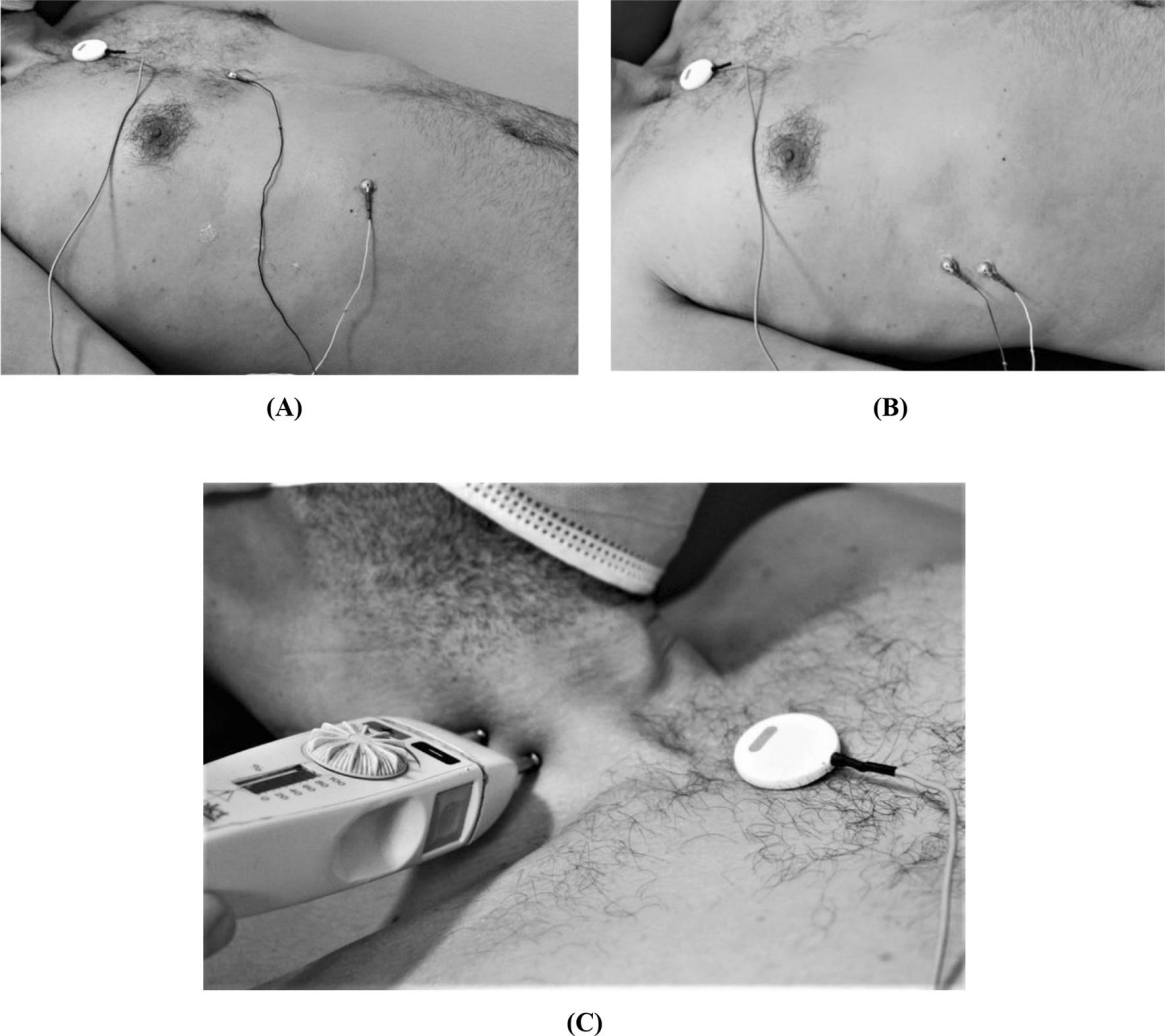
Position of the stimulating and recording electrodes of the phrenic nerve conduction study. (**A**) Sternal recording; active electrode was placed 5 cm superior to the tip of the xiphoid process and the reference was placed 16 cm from the xiphoid process at costal margin ipsilateral to the stimulation site. (**B**) LCW recording; active electrode over the 7th intercostal space at the anterior axillary line and the reference electrode at the 8th intercostal space ipsilateral to the stimulation site. (**C**) Position of the stimulating electrode at the posterior border of the sternocleidomastoid muscle at the level of the cricoid cartilage.

Phrenic nerve conduction study was performed bilaterally. Subjects were lying supine with the head slightly elevated and rotated to the opposite side to the nerve under stimulation. The phrenic nerve was supramaximally stimulated at the posterior border of the sternocleidomastoid muscle at the level of the cricoid cartilage with the anode placed proximal to the cathode, Fig. 1. Rectangular pulses of 0.2–0.5 ms duration were used. Measurements were made separately during normal inspiration and expiration. Co-stimulation of the brachial plexus was observed as indicated by muscle contractions, arm movement, paraesthesia (reported by the subject), a short latency < 5 ms, low amplitude and initially positive response. In such case, the cathode was repositioned medially to preferentially stimulate the phrenic nerve.

The DCMAP was recorded from over the sternum with the active electrode placed 5 cm superior to the tip of the xiphoid process and the reference electrode placed 16 cm from the xiphoid process at costal margin ipsilateral to the stimulation site (sternal recording). DCMAP was also recorded from over the lateral chest with the active electrode placed over the 7th intercostal space at the anterior axillary line and the reference electrode at the 8th intercostal space ipsilateral to the stimulation site (LCW recording), Fig. 1. Two responses were recorded at each site and the average values of the two responses were calculated.

The filter band width was set at 2 Hz to 10 kHz, sweep speed at 5 ms/division, and sensitivity at 200–2000 µV/ division. Measured parameters included the onset latency, amplitude (peak to peak), duration (negative phase), and area. Side-to-side differences were calculated for all parameters. Side-to-side amplitude difference was expressed as a ratio between the two sides.

Peripheral neuropathy was excluded electro-physiologically^31^.

### Statistical analysis

Data were analyzed using IBM SPSS software package version 20.0. (Armonk, NY: IBM Corp) The Kolmogorov–Smirnov test was used to verify the normality of distribution and all parameters were normally distributed. Quantitative data were described using range, mean, and standard deviation. Significance was judged at the 5% level. Cut-off values were calculated as mean ± 2SD. Paired t-test was used to compare between two periods. Student t-test was used to compare between two studied groups. Pearson coefficient was used to correlate between two normally distributed quantitative variables. Multivariate regression analysis was used to estimate the relationship between different dependent and independent variables.

### Ethics approval and consent to participate

The study was approved by the local ethics committee of the Faculty of Medicine, Alexandria University and the procedures used in this study adhere to the tenets of the Declaration of Helsinki. Informed consent was obtained from all individual participants included in the study.

## Results

The phrenic nerve could not be stimulated in 3 subjects (short/obese neck). Data presented are pooled data (140 nerves from right and left sides in 70 subjects). All parameters were normally distributed, so transformation of data was not required.

**Figure 2:**
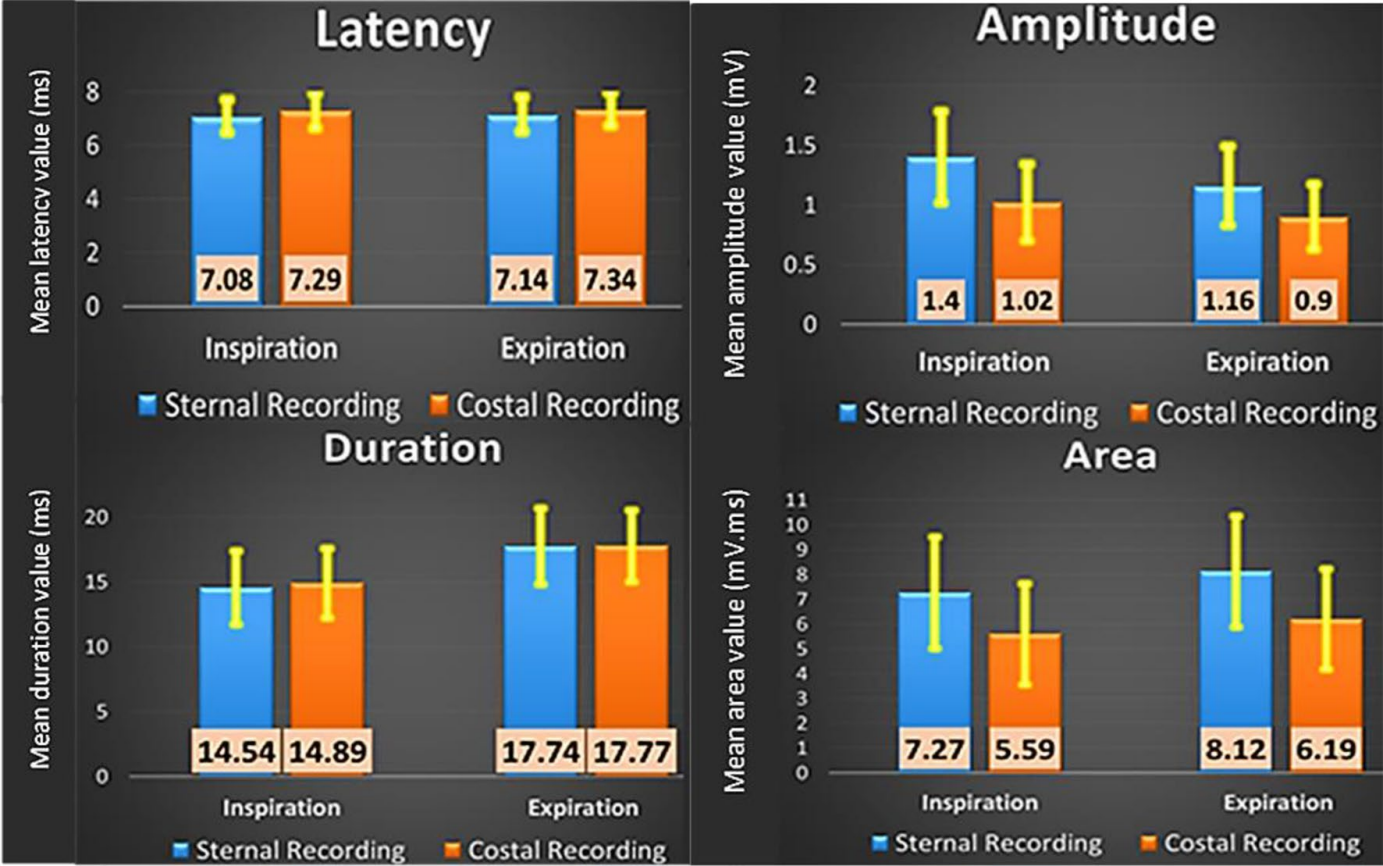
Mean values of sternal and LCW DCMAPs parameters during inspiration and expiration.

Mean, normative, and inter-side difference values of sternal and LCW potentials during inspiration and expiration are presented in Table 1. Mean values of sternal and LCW DCMAPs parameters during inspiration and expiration are illustrated in Fig. 2. The latency of sternal potentials was significantly shorter than LCW potentials with significantly higher amplitude and larger area both during inspiration and expiration (*p* < 0.001). The duration did not differ significantly between the two recording sites during inspiration and expiration (*p* = 0.138 and 0.919, respectively).

**Table 1:** The mean, range and normative- interside difference values of sternal and LCW DCMAP parameters during inspiration and expiration.

		Min.–Max	Mean ± SD	Limit values			
Latency (ms):	**Sternal DCMAP**						
	Inspiration	5.9–8.6	7.08 ± 0.63	8.34	0–0.9	0.31 ± 0.20	0.71 (10.6%)
	Expiration	5.7–9	7.14 ± 0.67	8.48	0–0.9	0.28 ± 0.21	0.7 (10.4%)
	**LCW DCMAP**						
	Inspiration	5.9–9.1	7.29 ± 0.67	8.63	0–0.7	0.29 ± 0.20	0.69 (10.3%)
	Expiration	5.9–9.6	7.34 ± 0.65	8.64	0–0.9	0.31 ± 0.21	0.73 (11.1%)
Amplitude (mV):	**Sternal DCMAP**						
	Inspiration	0.6–2.4	1.4 ± 0.4	0.60	0–35%	14 ± 10	34%
	Expiration	0.4–2.2	1.4 ± 0.4	0.46	0–36%	15 ± 10	35%
	**LCW DCMAP**						
	Inspiration	0.4–1.9	1.02 ± 0.34	0.34	0–43%	16 ± 11	38%
	Expiration	0.4–1.7	0.9 ± 0.29	0.32	0–43%	16 ± 10	36%
Duration (ms):	**Sternal DCMAP**						
	Inspiration	8.44–21.3	14.54 ± 3.14	20.82	0–5.9	2.27 ± 1.5	5.27 (30%)
	Expiration	11–26	17.74 ± 3.19	24.12	0–5.2	2.25 ± 1.4	5.15 (25.7%)
	**LCW DCMAP**						
	Inspiration	9.1–20.65	14.89 ± 2.96	20.81	0–5.3	2.21 ± 1.4	5.1 (29.97%)
	Expiration	10.4–24.1	17.77 ± 3.02	23.81	0–6	2.1 ± 1.5	5.1 (25.1%)
Area (mV.ms):	**Sternal DCMAP**						
	Inspiration	2.9–12.9	7.27 ± 2.4	2.47	0–6.4	1.24 ± 1.13	3.5 (31.6%)
	Expiration	2.3–13.5	8.12 ± 2.4	3.32	0–7.6	1.23 ± 1.29	3.81 (28.8%)
	**LCW DCMAP**						
	Inspiration	2.2–12.8	5.59 ± 2.13	1.33	0–3.3	1.07 ± 0.76	2.59 (33.5%)
	Expiration	2.6–13.4	6.19 ± 2.12	1.95	0–3.85	1.05 ± 0.82	2.69 (33.2%)

Values determined from 140 phrenic nerves in 70 normal subjects.

Normative values determined as mean ± 2SD.

Interside amplitude and area differences are expressed as the ratio between the two sides.

The sternal and LCW DCMAPs showed significantly shorter latency, shorter duration, higher amplitude and lower area during inspiration than during expiration, (*p* = 0.015, < 0.001, < 0.001, < 0.001, and 0.04, < 0.001, < 0.001, < 0.001, respectively).

The latency and duration were significantly longer on the right side (except for latency of sternal potentials during inspiration) whereas the amplitude was significantly higher on the left side (except for sternal potentials during expiration). The area did not differ between the two sides, Table 2. Parameters from right and left sides were strongly/very strongly correlated, Table 2. The mean latency, amplitude, and area were significantly higher in men. The duration did not differ significantly between the two sexes, Table 3.

**Table 2:** Comparison between right and left DCMAPs recorded from over the sternum and LCW during inspiration and expiration.

Parameter	Recording site (respiratory phase)	Right side	Left side	Paired t-test		Pearson coefficient	
				Test value	*P* value	r	*P* value
Latency (ms)	**Sternal DCMAP**						
	Inspiration	7.11 ± 0.62	7.05 ± 0.65	1.297	0.199	0.831	< 0.001*
	Expiration	7.20 ± 0.65	7.08 ± 0.69	3.103	0.003*	0.881	< 0.001*
	**LCW DCMAP**						
	Inspiration	7.36 ± 0.66	7.22 ± 0.68	3.618	0.001*	0.879	< 0.001*
	Expiration	7.41 ± 0.63	7.28 ± 0.67	3.044	0.003*	0.849	< 0.001*
Amplitude (mV)	**Sternal DCMAP**						
	Inspiration	1.36 ± 0.42	1.44 ± 0.38	2.516	0.014*	0.775	< 0.001*
	Expiration	1.16 ± 0.35	1.17 ± 0.35	0.415	0.679	0.747	< 0.001*
	**LCW DCMAP**						
	Inspiration	0.99 ± 0.31	1.05 ± 0.36	2.267	0.027*	0.783	< 0.001*
	Expiration	0.87 ± 0.27	0.93 ± 0.31	2.747	0.008*	0.804	< 0.001*
Duration (ms)	**Sternal DCMAP**						
	Inspiration	14.90 ± 3.15	14.17 ± 3.11	2.212	0.031*	0.641	< 0.001*
	Expiration	18.10 ± 3.42	17.38 ± 2.92	2.259	0.027*	0.680	< 0.001*
	**LCW DCMAP**						
	Inspiration	15.44 ± 2.97	14.33 ± 2.86	3.694	< 0.001*	0.659	< 0.001*
	Expiration	18.33 ± 3.20	17.20 ± 2.75	3.850	< 0.001*	0.696	< 0.001*
Area (ms.mV)	**Sternal DCMAP**						
	Inspiration	7.20 ± 2.31	7.35 ± 2.49	0.697	0.488	0.755	< 0.001*
	Expiration	8.14 ± 2.35	8.10 ± 2.47	0.180	0.858	0.725	< 0.001*
	**LCW DCMAP**						
	Inspiration	5.48 ± 2.06	5.71 ± 2.21	1.413	0.163	0.814	< 0.001*
	Expiration	6.16 ± 2.11	6.22 ± 2.16	0.374	0.709	0.802	< 0.001*

**Table 3:** Comparison between DCMAPs recorded in men and women (n = 70).

	Recording site (respiratory phase)	Men	Women	t-test	*P* value
Latency (ms)	**Sternal DCMAP**				
	Inspiration	7.21 ± 0.61	6.81 ± 0.59	3.7	< 0.001*
	Expiration	7.30 ± 0.62	6.83 ± 0.66	4.12	< 0.001*
	**LCW DCMAP**				
	Inspiration	7.49 ± 0.59	6.87 ± 0.63	5.761	< 0.001*
	Expiration	7.54 ± 0.59	6.95 ± 0.59	5.608	< 0.001*
Amplitude (mV)	**Sternal DCMAP**				
	Inspiration	1.56 ± 0.37	1.07 ± 0.22	8.227	< 0.001*
	Expiration	1.28 ± 0.34	0.91 ± 0.19	6.847	< 0.001*
	**LCW DCMAP**				
	Inspiration	1.11 ± 0.32	0.84 ± 0.31	4.782	< 0.001*
	Expiration	0.97 ± 0.28	0.76 ± 0.27	4.123	< 0.001*
Duration (ms)	**Sternal DCMAP**				
	Inspiration	14.90 ± 3.14	13.83 ± 3.05	1.871	0.063
	Expiration	17.85 ± 3.17	17.53 ± 3.24	0.549	0.587
	**LCW DCMAP**				
	Inspiration	15.32 ± 3.09	14.05 ± 2.52	1.859	0.068
	Expiration	18.00 ± 3.20	17.30 ± 2.61	1.252	0.213
Area (ms.mV)	**Sternal DCMAP**				
	Inspiration	8.23 ± 2.12	5.41 ± 1.72	7.62*	< 0.001*
	Expiration	8.98 ± 2.20	6.44 ± 1.84	6.554*	< 0.001*
	**LCW DCMAP**				
	Inspiration	6.28 ± 1.82	4.26 ± 2.07	5.696*	< 0.001*
	Expiration	6.71 ± 1.98	5.15 ± 2.04	4.155*	< 0.001*

*Statistically significant at *p* ≤ 0.05.

Table 4 shows the results of correlation analysis between DCMAP parameters and subjects’ characteristics. The latency of diaphragmatic potentials correlated positively with age (inspiration/expiration), height (inspiration/expiration), and chest circumference (sternal potentials during inspiration). The amplitude of sternal and LCW potentials (during inspiration/expiration) correlated negatively with the BMI and that of LCW potentials (inspiration) correlated negatively with chest circumference.

**Table 4:** Correlation between phrenic nerve conduction parameters and subjects’ characteristics.

		Age (years)		Height (m)		BMI (kg/m^2^)		Chest circumference (cm)	
		r	*p*	r	*p*	r	*p*	r	*p*
Latency	**Sternal DCMAP**								
	Inspiration	0.622	< 0.001*	0.335	0.005*	0.046	0.705	0.319	0.007*
	Expiration	0.590	< 0.001*	0.357	0.002*	0.042	0.732	0.288	0.016*
	**LCW DCMAP**								
	Inspiration	0.435	< 0.001*	0.436	< 0.001*	− 0.156	0.197	0.133	0.272
	Expiration	0.413	< 0.001*	0.415	< 0.001*	− 0.113	0.353	0.144	0.236
Amplitude	**Sternal DCMAP**								
	Inspiration	0.053	0.666	0.157	0.194	− 0.425	< 0.001*	− 0.146	0.227
	Expiration	0.082	0.498	0.077	0.524	− 0.288	0.016*	− 0.004	0.972
	**LCW DCMAP**								
	Inspiration	− 0.017	0.888	0.009	0.944	− 0.344	0.004*	− 0.239	0.047*
	Expiration	0.060	0.623	− 0.048	0.696	− 0.272	0.023*	− 0.193	0.109
Duration	**Sternal DCMAP**								
	Inspiration	− 0.004	0.976	0.313	0.011*	− 0.323	0.009*	− 0.204	0.103
	Expiration	− 0.136	0.282	0.201	0.108	− 0.394	0.001*	− 0.358	0.003*
	**LCW DCMAP**								
	Inspiration	− 0.079	0.532	0.358*	0.003*	− 0.162	0.196	0.066	0.601
	Expiration	− 0.079	0.534	0.375*	0.002*	− 0.153	0.223	0.030	0.815
Area	**Sternal DCMAP**								
	Inspiration	− 0.051	0.688	0.319	0.010*	− 0.545	< 0.001*	− 0.310	0.012*
	Expiration	− 0.016	0.902	0.270	0.010*	− 0.447	< 0.001*	− 0.210	0.094
	**LCW DCMAP**								
	Inspiration	− 0.062	0.626	0.179	0.155	− 0.365	0.003*	− 0.178	0.157
	Expiration	0.002	0.986	0.125	0.322	− 0.244	0.050*	− 0.144	0.253

r, Pearson coefficient.

*Statistically significant at *p* ≤ 0.05.

The duration of the sternal potentials correlated positively with height (inspiration) and negatively with BMI (inspiration/expiration) and chest circumference (expiration). The duration of the LCW potentials correlated positively with height (inspiration/expiration).

The area of sternal and LCW potentials correlated negatively with the BMI (inspiration/expiration) and that of sternal potentials correlated positively with height (inspiration/expiration).

Multiple regression analyses were done between different parameters of DCMAP (dependent variables) and subjects’ criteria (independent variables), Table 5.

**Table 5:** Multivariate analysis for the parameters affecting DCMAP parameters.

	Recording site	Respiratory phase		Unstandardized Coefficients		Standardized Coefficients	Test of sig	*p* value
				B	Std. Error	Beta		
Latency	Sternal DCMAP	Inspiration	(Constant)	3.495	1.613		2.167	0.034
			Age	0.029	0.005	0.556	6.048	0.000*
		Expiration	(Constant)	3.473	1.761		1.972	0.053
			Age	0.030	0.005	0.528	5.608	0.000*
	LCW DCMAP	Inspiration	(Constant)	4.390	1.750		2.508	0.015
			Age	0.022	0.005	0.384	3.973	0.000*
			Gender	− 0.419	0.166	0.305	2.530	0.014*
		Expiration	(Constant)	4.922	1.716		2.869	0.006
			Age	0.020	0.005	0.365	3.685	0.000*
			Gender	0.414	0.163	0.315	2.549	0.013*
Amplitude	Sternal DCMAP	Inspiration	(Constant)	2.329	0.183		12.745	0.000
			Gender	0.432	0.083	0.543	5.227	0.000*
		Expiration	(Constant)	1.732	0.174		9.967	0.000
			Gender	0.351	0.079	0.511	4.470	0.000*
	LCW DCMAP	Inspiration	(Constant)	2.819	0.538		5.239	0.000
			Gender	0.354	0.101	0.524	3.507	0.001*
			Chest circumference	− 0.017	0.007	− 0.406	− 2.335	0.023*
		Expiration	(Constant)	1.322	0.162		8.166	0.000
			Gender	− 0.167	0.073	0.287	2.276	0.026*
Duration	LCW DCMAP	Inspiration	(Constant)	4.339	6.328		0.686	0.495
			Height	11.415	3.753	0.358	3.042	0.003*
		Expiration	(Constant)	3.008	6.484		0.464	0.644
			Height	12.334	3.845	0.375	3.208	0.002*
Area	Sternal DCMAP	Inspiration	(Constant)	22.798	5.887		3.873	0.000
			Gender	3.119	0.621	0.661	5.019	0.000*
			Chest circumference	− 0.115	0.045	− 0.402	− 2.570	0.013*
		Expiration	(Constant)	19.644	6.555		2.997	0.004
			Gender	2.301	0.627	0.490	3.671	0.001*
			BMI	− 0.120	0.050	− 0.276	− 2.400	0.019*
	LCW DCMAP	Inspiration	(Constant)	9.803	1.146		8.556	0.000
			Gender	− 1.651	0.518	− 0.388	3.186	0.002*
		Expiration	(Constant)	9.068	1.225		7.404	0.000
			Gender	− 1.344	0.554	− 0.317	− 2.425	0.018*

Latency of sternal and LCW potentials (inspiration/expiration) was related to age (*p* < 0.001). Latency of LCW potentials (inspiration/expiration) and amplitude and area of sternal and LCW potentials (inspiration/expiration) were related to gender. Amplitude of LCW potentials and area of sternal potentials (inspiration) were related to chest circumference (*p* = 0.023 and 0.013 respectively). Area of sternal responses (expiration) was related to the BMI (*p* = 0.019). Duration of LCW responses (inspiration/expiration) was related to height (*p* = 0.003 and 0.002 respectively).

**Figure 3:**
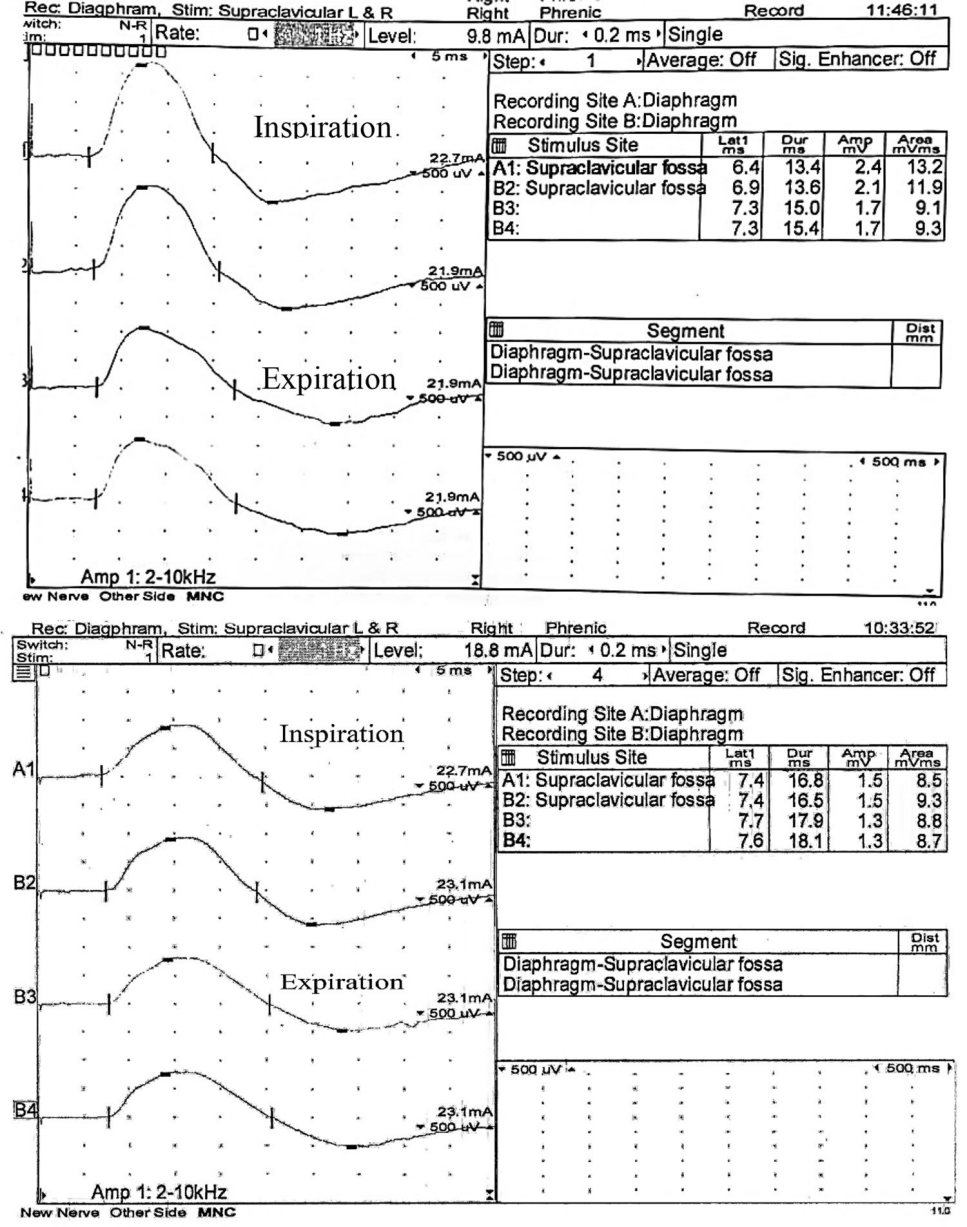
DCMAPs recorded from over the sternum and LCW in one of the studied subjects showing shorter latency and duration, higher amplitude, and area in sternal compared to LCW recordings.

An example of DCMCPs recorded from over the sternum and lateral chest wall in one of the studied subjects is shown in Fig. 3.

## Discussion

Overall, our mean and normative values of DCMAPs parameters were comparable to those reported in the literature. Differences are mostly related to variations among studies in the site of stimulation or signal recording or how the parameter was measured.

The mean latency values of sternal potentials [7.08 ms (inspiration) and 7.14 ms (expiration)] are higher than values reported by Chen et al. (6.5 ms)^15^, Maranhão et al. [6.12 (inspiration) and 6.42 ms (expiration)]^17^, Resman-Găspěrsč and Podnar [6.55 (inspiration) and 6.59 ms (expiration)]^16^, Vincent et al. (6.59 ms)^18^, and Dionne et al. (6.6 ms)^20^, They all stimulated the nerve at a lower level (supraclavicular fossa).

The mean latency values of LCW potentials [7.29 ms (inspiration) and 7.34 ms (expiration)] are close to values reported by Luo et al. (7.3 (left side) and 7.6 ms (right side)^25^, Dionne et al. (7.14 ms)^20^, and Mckenzie and Gandevia [7.68 (right side) and 7.92 ms (left side)]^22^. They adopted the same stimulation and recording montages as the current work.

Our upper limits of the latency [8.34 (sternal/inspiration), 8.48 (sternal/expiration), 8.63 (LCW/inspiration), 8.64 ms (LCW/expiration)] are comparable to values by Vincent et al. [(8.36 ms (sternal)]^18^ and Resman- Găspěrsč and Podnar [7.92 ms (sternal/inspiration and expiration)]^16^. They are higher than values reported by Maranhão et al. (6.34 ms (sternal/inspiration) and 6.42 (sternal/expiration)^17^. Chen et al. found that the suggested normal latency limit is < 8 ms (sternal)^15^. Variation in limit values among studies is again related to variation in stimulation and recording sites.

Our mean amplitude values are higher than values reported in the literature^15–20,22^. This is because we measured the amplitude from peak-to-peak as opposed to other studies where the amplitude was measured from onset-to-peak. Highest mean amplitude values were recorded from over the sternum (1.4 (inspiration) and 1.16 mV (expiration). Lower limits of the amplitude [0.60 (sternal/inspiration), 0.46 (sternal/expiration), 0.34 (LCW/inspiration), 0.32 mV (LCW/expiration)] are comparable to values by other studies (range: > 0.3–0.7 mV)^15,16^. Swenson and Rubenstein^19^, Johnson et al.^32^, and Vincent et al.^18^ on the other hand reported much lower normative values [0.1, 0.12, and (0.14 right and 0.11 left) mV respectively). As illustrated by Maranhão et al.^17^, the wide range of phrenic nerve amplitude creates a great problem in determining a lower normal limit.

Mean and normative values of the duration of sternal DCMAP in the current work [14.54 and 20.82 ms (inspiration) and 17.74 and 24.12 ms (expiration)] are within the range of values reported by Chen et al.^15^, Resman- Găspěrsč and Podnar^16^, Maranhão et al.^17^, and Vinvent et al.^18^ They all recorded the potentials from over the sternum. No data is available in the literature on the normative value of the duration of potentials recorded from over the LCW.

Mean values of the area of sternal DCMAP [7.27 mV.ms (inspiration) and 8.12 mV.ms (expiration)] are close to values in the studies by Chen et al.^15^, Resman-Găspěrsč and Podnar^16^, and Maranhão et al.^17^ Our normative values [2.47 mV.ms (inspiration) and 3.32 mV.ms (expiration)] are however lower than other studies (lower limit value > 4 mV.ms)^15–17^. This variation is mostly related to how the limit normal was determined (mean-2SD as in the current work compared to 5th percentile limit in other studies). Our values are higher than that by Vincent et al.^18^ (mean = 3.05 mV.ms and limit normal = 0.87 mV.ms). Lower amplitude values have contributed to lower area values in their study.

The means values of the area of DCMAP from over LCW ([5.59 mV.ms (inspiration) and 6.19 mV.ms (expiration)] are comparable to value by Dionne et al. (6.41 mV.ms)^20^. Data on the normative values from costal diaphragm is scanty in the literature.

Our mean and normative values of the interside differences in latency, amplitude, duration, and area were presented. These values are useful in assessment of patients with a unilateral phrenic nerve lesion as those caused by trauma or surgery especially when the absolute values of both sides are within the normal range. Our values are consistent with those in the studies by Chen et al.^15^ and Resman-Găspěrsč and Podnar^16^. Vincent et al.^18^ reported higher interside differences in the latency, amplitude, and duration of potentials recorded from over the sternum. Swenson and Rubenstein^19^ also reported higher mean interside amplitude differences of potentials recorded from over the sternum and LCW which may be related to differences in the placement of recording electrodes.

### Differences between sternal and LCW DCMAPs

Comparative studies showed that the latency, amplitude, and area varied significantly between sternal and LCW potentials. The duration did not differ between the two recording sites.

The latency of sternal potentials was significantly shorter than LCW potentials. Swenson and Rubenstein^19^ reported similar finding. This can be expected considering the shorter length of the anterior (sternal) branch. McKenzie and Gandevia^22^ in their illustrative work provided the distance from the branching points of phrenic nerve to different recording sites, which should correspond to the length of different branches. Distance corresponding to the anterior branch was shorter than that for lateral branch on the right (6.7 and 13.8 cm) and left sides (2.8 and 8.8 cm respectively).

The amplitude of sternal potentials was significantly higher than LCW potentials. The finding is consistent with that by Dionne et al.^20^ who recorded diaphragmatic potentials from over six different sites. The highest amplitude was obtained from over the sternum. They related this finding to the orientation of the electrical dipole of the diaphragm (different from limb muscles). They assumed that G1 and G2 are both active, with the DCMAP representing out-of-phase summation of opposite polarity activity at the two electrodes, and this in part has accounted for larger amplitude at the sternum as it was the only technique with G1 positioned above the xiphoid.

Amplitude difference between sternal and LCW potentials can also be due to variation between the two sites in the distance between the active and reference recording electrodes^33^ with higher amplitude of sternal potentials being related to longer distance between recording electrodes compared to LCW recording site (16 and 3.5–5 cm respectively).

Recording of DCMAP from over the sternum was recommended by Chen et al.^15^ and Dionne et al.^20^ as this electrode position gives the maximum amplitude. It also allows the easiest and most rapid study to perform as it does not involve rib counting or multiple electrodes repositioning especially in obese subjects and in patients in intensive care unit where chest tubes and catheters are frequently encountered.

Contrary to our finding, Swenson and Rubenstein^19^ found smaller but easily recorded potentials at the sternum. Maximum peak was recorded over the anterolateral chest at the intersection of the axillary line with transverse plane through the xiphoid. In their study both xiphoid and costal recordings had their reference electrodes placed below the umbilicus which may have accounted for their results.

The duration did not differ significantly between sternal and LCW potentials. This parameter reflects the range of conduction velocities of conducting nerve fibers^34^ and is not expected to differ between DCMAPs recorded from over the two sites. Significantly larger mean area of sternal potentials is consistent with significantly higher amplitude values of sternal compared to LCW potentials with no difference in the duration between them.

Despite the given advantages of recording from over the sternum (higher amplitude and easier study with no need for rib counting or repositioning of the electrodes)^15,20^, it cannot be concluded that this montage should be the standard one for recording DCMAPs.

The diaphragm is a specialized muscle that demonstrates distinct muscular subvolumes (neuromuscular compartments) in which the intramuscular phrenic nerve distribution (branching) is confined^29^. Recording electrodes at each recording site are relatively selective for the subjacent portion and record activity from the underlying portion of the diaphragm^22^ that is innervated by separate nerve branches^29^.

The presence of significant differences between potentials recorded from over the sternum and lateral chest wall in the current work and in other studies^19,20,22^ as well as the evidence by some electrophysiological studies that one branch but not the other can be affected in different medical conditions^35,36^ highlights the importance of assessing the conduction along different nerve branches in every case referred for electrophysiological evaluation. It also highlights the need to provide normative data for potentials recorded from different diaphragm subvolumes innervated by different branches of the nerve.

### Differences between DCMAPs recorded during inspiration and expiration

We found variation in DCMAP parameters with respiratory cycle. The mean latency, duration and area were significantly lower, and the mean amplitude was significantly higher during inspiration compared to expiration. Findings are mostly related to changing lung volumes during movement of the diaphragm in the respiratory cycle and changing physiological properties of the diaphragm during contraction^15,37^.

Similar to our finding, Maranhão et al.^17^ found shorter latency of DCMAPs during inspiration. Resman-Găspěrsč and Podnar^16^ found significant difference in peak but not the onset latency between inspiration and expiration. DCMAPs were recorded from over the sternum in both studies. Latency of CMAP represents the summated durations of impulse propagation along the nerve fiber, time delay across the neuromuscular junction, and depolarization time across the muscle^31^. Increased muscle fibers conduction velocity during contraction (inspiration) therefore decreases the overall time needed for impulse propagation and hence decreases the response latency^38^.

Significantly higher amplitude, shorter *duration* and smaller area during inspiration come in agreement with the results of different studies. Resman-Găspěrsč and Pondar^16^ found similar changes in the amplitude and duration during inspiration. Changes in the area were however insignificant. Chen et al.^15^ reported increase in the amplitude and decrease in the duration with inspiration. Maranhão et al.^17^ demonstrated the same results although they did not conduct a statistical comparison. On the other hand, Swenson and Rubenstein^19^ reported higher DCMAP amplitude during expiration. They recorded the potentials at the level of the 7th intercostal space with the reference electrode below the umbilicus.

The amplitude value of diaphragmatic potentials depends on the orientation of recording electrodes and the distance between recording electrodes and the diaphragm which change during the respiratory cycle^15^. Chen et al. suggested that during inspiration the diaphragm flattens which changes the angle, the moving dipole meets at the recording electrodes^15^ which according to the theory of volume conduction would result in increased CMAP amplitude^39^. Resman-Găspěrsč and Pondar added that shortening (shorter path) and thickening (faster conduction) of diaphragmatic muscle during contraction (inspiration) make larger **amplitude** and shorter conduction time and shorter duration^16^.

In a similar context, it was proved by musculoskeletal ultrasound that thickness of the diaphragm in healthy individual increases by 28–96% during inspiration relative to expiration^40,41^. This in turn increases conduction velocity of muscle fibers due to diminished resistance of thick fibers and improves synchronization of the electrical potentials. In addition, muscle fibers shortening bring them closer to the recording electrodes, thus muscle contraction increases temporal and spatial summation of muscle fibers action potentials leading to increased CMAP amplitude and decreased duration^42^**.**

We found significantly smaller area during inspiration due to significant decrease in the duration that overweighed the increase in amplitude during inspiration. Maranhão et al.^17^ demonstrated decrease in the area of DCMAPs during inspiration. They did not however conduct a statistical comparison. Resman-Găspěrsč and Pondar^16^ found that difference in the area between inspiration and expiration is not significant, still smaller during inspiration. They considered the area to be more useful than the amplitude due in part to its insensitivity to respiratory cycle. The current study was conducted on larger number of healthy individuals (70 subjects) compared to that by Resman-Găspěrsč and Pondar^17^ (29 healthy subjects). It is evident that there is an assent among studies that the area of DCMAPs is smaller during inspiration. The variation is about the extent of the difference whether it is significant or not. The exact change of the area with respiration is to be further evaluated.

### Differences between DCMAPs recorded on right and left sides

We found significantly longer latency of diaphragmatic potentials on the right side. Mier et al.^2^ reported similar finding but did not mention an explanation. Delhez et al.^43^ and Katayama et al.^44^ on the other hand showed longer latency on the left side. They attributed their finding to longer anatomical course of the left phrenic nerve. Maranhão et al.^17^, Mckenzie and Gandevia^22^, Vincent et al.^18^ and Chen et al.^15^ did not find significant differences between both sides.

The course as well as the length of the right and left phrenic nerves varies. Jiang et al. measured the full length and the length of thoracic part of the phrenic nerve on both sides and both were shorter on the right side. The intramuscular branches were however longer on the right side in higher number of their studied corpses^45^. McKenzie and Gandevia measured the conduction distance from entry point of phrenic nerve branches into the diaphragm to motor points adjacent to different recording sites, which should correspond to length of intramuscular branches. All distances were longer on the right side^22^. This can be expected given that the right phrenic nerve has almost as straight course. It enters the diaphragm close to the esophageal hiatus^29,45^, thus at a point at longer distances from the anterior and lateral chest walls than the left phrenic nerve. The conduction along these branches (thin) is slower than conduction along the nerve trunk^31^. Both factors (the length and conduction velocity along the nerve trunk and the branches) may have accounted for the longer latency of the recorded potentials on the right side.

The amplitude of DCMAPs (except for sternal potentials during expiration) was significantly lower on the right side. Resman-Găspěrsč and Podnar similarly found lower amplitude of sternal potentials on the right side during inspiration but not expiration and attributed the finding to the higher position of right hemidiaphragm, which is lifted by the liver (longer and thinner muscle fibers)^16^. Absence of a difference between right and left sternal potentials during expiration can be expected because at the xiphoid and during expiration, anatomical and physiological differences between the two sides are kept to minimum.

The duration of diaphragmatic potentials was significantly longer on the right side. Given that electrical potentials can propagate easily through thickened muscle fibers with lower resistance, (Kim BJ) it can be speculated that the duration of DCMAPs from the right hemidiaphragm (thinner fibers) is longer than the left. The area did not differ between the right and left sides. Similar findings were found by Resman-Găspěrsč and Podnar^16^. This is quite expected as lower amplitude and higher duration on the right side are equated by higher amplitude and lower duration on the left side.

Despite the difference in DCMAP parameters between the right and left sides, we found strong/highly strong right-to-left correlation in all parameters (*p* < 0.001) indicating that if one side deviated from the mean value the other side tended also to deviate in the same direction. Mier et al. also found significant correlation between right and left phrenic nerve conduction time (r = 0–81, *p* < 0–001)^3^. Swenson and Rubenstein found constancy of only the onset latency. They did not find right-to-left amplitude correlation^19^. Similar to their findings, Maranhão et al. did not find consistent right-to-left correlation^17^. The presence of strong/highly strong right-to-left correlation indicates that in unilateral lesion, the opposite side would serve as a reasonable standard for comparison.

### Differences between DCMAPs recorded in men and women

Measured parameters were different between men and women. The mean latency, amplitude, and area of DCMAPs were significantly increased in men. The duration did not differ significantly between both sexes. Maranhão et al. reported substantial differences in amplitude, latency, and duration of DCMAPs between men and women^17^. Resman-Găspěrsč and Pondar^16^ and Vincent et al.^18^ found significantly higher amplitude in men. Vincent et al.^17^ found that the latency was different after adjustment for age and BMI.

Differences between men and women in parameters of DCMAPs are mostly related to variations in the anthropometric measures between both sexes (longer nerves contributing to longer latency and greater muscle mass contributing to higher amplitude in men)^16,18^. Larger area in men is due to significantly higher amplitude and longer duration (not to significant level) in men compared to women. Only the **duration** of the DCMAPs did not differ significantly between men and women. This finding is expected given that the duration reflects range of conduction velocities of nerve fibers^34^ which should not differ by gender.

### The correlation and regression analyses between different parameters of phrenic nerve conduction study and subjects’ demographic and anthropometric data

Correlation and regression analyses revealed that latency of DCMAPs is significantly related to age which is consistent with data from several studies^15–18^ and can be attributed to dropout of largest fibers, segmental degeneration, and reduced internodal length with aging^46,47^. No correlation was found between *age* and other parameters. The same was reported by Maranhão et al.^17^ and Vincent et al.^18^ and can be explained by the exclusion of elderly individuals from enrollment in the study (oldest individual was 61 years). Prominent electrophysiological changes in amplitude, duration, and area are seen in individuals above the age of 60–65 years^48^.

Regression analysis showed significant relation between amplitude and area of DCMAPs and gender. The finding is consistent with the presence of significant differences between men and women in the amplitude and area of DCMAPs and can be explained by larger muscle mass in men. The latency of LCW (but not sternal) potentials was also related to gender. The contribution is indirect and is in part related to longer nerves in men (more evident for the lateral branch).

The latency of DCMAPs was also found to correlate with height which can be explained by increased length of nerves with relative conduction slowing in tall individuals^31^. Our results agree with Resman-Găspěrsč and Podnar^16^ and Maranhão et al.^17^ Results of the regression analysis did not however show a relation between latency of DCMAPs and height. This indicates that height is not an independent contributor to the latency of DCMAPs. McKenzie and Gandevia found that conduction distance does not directly relate to the subject’s height and conduction velocity is not uniform along the main nerve trunk^22^. In one study, the length of the sternum was used as a surrogate for the height. Nevertheless, it was not found to correlate with the latency of DCMAPs^16^. It is evident that the length of the phrenic nerve cannot only be represented by subjects’ height as the intramuscular branches which vary in thickness and size also contribute to the conduction distance and latency.

The duration of DCMAPs correlated with height. This is because relative conduction slowing in tall individuals decreases the synchronization of muscle fibers potentials which increases the duration of CMAP^49^. This is further supported by the results of the regression analysis where duration of only the LCW recordings was found to be related to height. Lateral intramuscular branch is longer than sternal (anterior) branch^22^ which should magnify the effect of increased length on the duration of the potentials.

The amplitude and area of sternal and LCW potentials (during inspiration and expiration) correlated negatively with the BMI. This is most likely due to amplitude attenuation by the thicker subcutaneous tissue in the person with higher BMI^50,51^. Such a relation was not shown in the results of regression analysis which indicate that BMI is not an independent contributor to amplitude and area of the DCMAPs yet stills a factor. Factors as the mass of the diaphragm (not measured in the current work) may have major contribution to the amplitude and area than the BMI.

Absence of a correlation between the latency of DCMAPs and BMI is plausible since the latency represents the conduction along the fastest fibers regardless the number of axons^31^. Thus in clinical setting, the fastest fibers appear to conduct equally in thin and obese individuals.

Chest circumference did not correlate with most of the recorded parameters. It only correlated positively with the latency of sternal potentials during inspiration and expiration and negatively with amplitude (of LCW DCMAP) and area (of sternal DCMAP) during inspiration and duration of sternal potentials during expiration. Multiple regression analysis revealed that BMI is related to the amplitude of LCW responses and the area of sternal responses during inspiration (*p* = 0.023 and 0.013 respectively).

Our results are contradictory to that by Resman-Găspěrsč and Podnar^16^ who found significant positive correlation between chest circumference and the CMAP area and Chen et al.^15^ who found significant positive correlation between chest circumference and the amplitude of diaphragmatic potentials and attributed this to the greater diaphragmatic muscle mass and more flattened diaphragms in persons with larger chest circumference. These contradictory findings may be because we measured the chest circumference at the level of the 4th intercostal space while Chen et al.^15^ measured the circumference at the level of the xiphoid process. Also, the intervening breast tissue in women may have accounted for differences in the measures of chest circumference between the two levels. The rib cage cross-section area may be a better measure to correlate with DCMAPs.

### Technical aspects

We stimulated the nerve at the posterior border of the sternocleidomastoid muscle at the level of cricoid cartilage. This stimulation site is easy to locate, less painful and it is easy to avoid brachial plexus co-stimulation by moving the electrode more medial^2,25,52^. It is to be mentioned that, the stimulation site can vary from subject to subject and may even vary between the left and the right sides of one subject due to asymmetry^16^.

The phrenic nerve was generally easy to stimulate. Difficulties, especially on the left side, were sometimes encountered possibly due to anatomical variation of the phrenic nerve that may pass through the anterior scalene muscle or there may be medial or lateral displacement of the phrenic nerve^53^. This difficulty was overcome by increasing current intensity and/or medial repositioning of the stimulating electrode. In 3 subjects with short or obese neck the nerve could not be stimulated (despite more rotation and/or extension of the neck was tried). In clinical practice, if percutaneous electric stimulation could not be performed due to technical problems, needle electric stimulation or magnetic stimulation of the phrenic nerve is to be tried^24,26^.

In the current study only the peak-to-peak amplitude was measured (for more precise measurement) and reported. This point is considered one of the study limitations.

## Conclusions and recommendations

The results of the current work showed that there are notable differences in the parameters of DCMAP between the sternal and LCW sites, inspiration and expiration, right and left sides, and men and women.

Normative values of diaphragmatic potentials along different branches should be provided. Moreover, the effect of age, gender, height, and BMI should be considered. The technique should be standardized regarding phases of respiration and the identification of the parameters to be measured. This is essential for valid comparison between patients and healthy subjects or during follow up and for comparisons between different labs and different studies.
